# END-OF-LIFE HEALTH LITERACY: VALIDATION STUDY OF A NEW INSTRUMENT, THE END-OF-LIFE HEALTH LITERACY SCALE (EOL-HLS)

**DOI:** 10.1093/geroni/igac059.1023

**Published:** 2022-12-20

**Authors:** Clément Meier, Sarah Vilpert, Gian Domenico Borasio, Ralf J Jox, Jürgen Maurer

**Affiliations:** University of Lausanne, Lausanne, Vaud, Switzerland; University of Lausanne, Lausanne, Vaud, Switzerland; Lausanne University Hospital and University of Lausanne, Lausanne, Vaud, Switzerland; Lausanne University Hospital and University of Lausanne, Lausanne, Vaud, Switzerland; University of Lausanne, Lausanne, Vaud, Switzerland

## Abstract

Measuring health literacy allows to assess individuals’ competencies to deal with health issues; it influences how individuals perceive their health problems, communicate with healthcare providers, or make medical decisions. The end of life is commonly characterized by one or several diseases, healthcare services’ uses and requires individuals to make complex medical decisions. Although the end-of-life concerns everyone, the level of competencies of individuals to get through this stage of life has been little explored. This study aims to fill this gap by validating a new instrument, the End-of-life Health Literacy Scale (EOL-HLS), in a representative sample of older adults aged 58+ living in Switzerland. We use the Swiss wave 8 (2019/2020) of SHARE. Based on the seminal work of Nutbeam (2000), end-of-life health literacy skills are measured using questions on the difficulty in understanding medical interventions, finding information, communicating, deciding in advance, and choosing end-of-life care options. In addition, we compare the findings to the European Health Literacy Survey questionnaire (HLS-EU-Q16). The results confirmed the suitability for performing factor analysis (KMO = 0.924, Bartlett’s test of sphericity statistically significant), a three-factor model was established and showed good fit properties (CFI = 0.964, TLI = 0.958, RMSEA = 0.047, SRMR = 0.067) and good reliability (α = 0.93). The associations found between individuals’ sociodemographic characteristics and the HLS-EU-Q16 were also present in our instrument, but higher EOL-HLS scores were associated with more positive end-of-life outcomes. The EOL-HLS is a reliable and valid instrument to target individuals with low end-of-life health literacy.

